# The Saharan Oscillation Index (1950–2024): Statistical Characterization, Persistence, and Spectral Variability

**DOI:** 10.21203/rs.3.rs-10768050/v1

**Published:** 2026-10-28

**Authors:** Younes TEBBAAI, Kenza KHOMSI, Mohamed KHALIS

**Affiliations:** 1 Department of Public Health and Clinical Research, Mohammed VI Center for Research and Innovation, Rabat, Morocco; 2 Mohammed VI International School of Public Health, Mohammed VI University of Sciences and Health, Casablanca, Morocco; 3 Higher Institute of Nursing Professions and Health Techniques, Ministry of Health and Social Protection, Rabat, Morocco

**Keywords:** Saharan Oscillation Index, atmospheric circulation, climate variability, North Africa, temporal variability, spectral analysis, climatology

## Abstract

This study presents a comprehensive statistical and spectral characterization of the Saharan Oscillation Index (SaOI), an atmospheric circulation metric describing the standardized sea-level pressure difference between the Azores High and the Saharan depression over the 1950–2024 period. Using monthly SaOI values derived from ERA5 reanalysis, we assessed distributional properties, temporal persistence, spectral variability, time-frequency structure, and long-term trends using complementary time-series methods. The SaOI exhibits a near-Gaussian central distribution (μ = 0.1954, σ = 0.1137). Although the original monthly series shows moderate lag-1 autocorrelation (r ≈ 0.50), lag-1 autocorrelation is substantially reduced after removal of the climatological seasonal cycle (r_1_ = 0.128), indicating weak residual month-to-month dependence. Fourier and wavelet analyses identify the annual cycle as the dominant component of variability, with a weaker semi-annual component, whereas no distinct persistent narrow-band interannual oscillation is evident. A weak positive long-term trend is detected (Sen’s slope = 0.0004 units yr^−1^), but it is not statistically significant after accounting for serial dependence (p ≈ 0.17). These results establish a reproducible statistical baseline for interpreting SaOI variability and provide a foundation for subsequent investigations of its teleconnections and regional climate impacts.

## INTRODUCTION

Climate change and air pollution are among the most pressing challenges of our time, with far-reaching implications for society (Afifa et al., 2024; Ofremu et al., 2024). The interactions between these environmental issues are particularly evident through atmospheric processes and meteorological conditions(Abbass et al., 2022; Bolan et al., 2024). Notably, air pollution is not confined to local sources only but is also influenced by large-scale transport mechanisms, leading to regional background pollution and heightened pollutant concentrations (Bolan et al., 2024; Chaurasia & Mohan, 2022; Qu et al., 2024).

The World Health Organization (WHO) underscores the severity of air pollution’s impact, estimating that it causes 7 million premature deaths globally each year (Lelieveld et al., 2015). Of particular concern is the exposure to suspended particulate matter (PM) in ambient air, which is a major contributor to this global health burden, with recent studies quantifying the mortality specifically linked to fine particle pollution(Vohra et al., 2021).

Air pollution, especially by particulate matter whose diameter is less than 10μm (PM10), is significantly impacted by meteorological factors, as extensively studied in prior research (Birim et al., 2023; Girotti et al., 2025). Specific synoptic patterns, often characterized by stagnant conditions with high relative humidity, thermal inversions, and low wind speeds, are strongly associated with hazardous PM10 incidents (Khomsi et al., 2020; Zhang et al., 2018).

Atmospheric circulation, a fundamental phenomenon shaping weather patterns and climate, operates in tandem with ocean circulation to redistribute thermal energy (Goudie & Middleton, 2001). Large-Scale Climate Indicators (LSCIs) are key metrics representing atmospheric and oceanic variability over broad spatial and temporal scales. They help analyze climate patterns, teleconnections, and their impacts on regional and global climates (Khomsi et al., 2024).

Undoubtedly, the Mediterranean region confronts notable environmental adversities such as droughts, flash floods, recurrent heatwaves, and desert dust and sandstorms (Middleton & Kang, 2017). Consequently, the necessity for early warning systems, in such regions, emerges as imperative to predict and manage these climate-induced calamities (Agbehadji et al., 2023; Islam et al., 2025). The integration of LSCIs into these systems holds promise in augmenting our capacity to anticipate extreme weather phenomena(White et al., 2017).

The atmospheric circulation over Northwest Africa is dominated by the seasonal interaction between two major pressure systems: the quasi-permanent Azores High, representing the northeastern extension of the subtropical North Atlantic anticyclone maintained by descending motion in the Hadley circulation (Rodwell & Hoskins, 2001), and the Saharan Heat Low, a thermally-driven low-pressure system developing over the Sahara-Sahel region during boreal summer through intense surface heating and deep boundary layer convection (Lavaysse et al., 2009). The meridional pressure gradient between these systems is an important control on northeasterly flow, subsidence over the Sahel, and the northward penetration of monsoon moisture from the tropical Atlantic. (Sultan & Janicot, 2003). Enhanced pressure gradients, associated with intensified Azores High and/or weakened Saharan Low, promote stronger subsidence, reduced moisture transport, and drought conditions (Nicholson & Webster, 2007). Conversely, weakened gradients facilitate enhanced monsoon flow and increased precipitation(Biasutti, 2019; Sultan & Janicot, 2003).

Recent advances in atmospheric reanalysis products, particularly the ERA5 dataset providing hourly global atmospheric fields at 31 km horizontal resolution from 1950-present with assimilation of extensive observational networks, enable robust quantification of historical pressure patterns over data-sparse regions including North Africa (Hersbach et al., 2020).

The (SaOI), previously introduced by Khomsi et al. (2020), captures the dynamics between the Azores High and Niamey Low (Khomsi et al., 2020). Its phase structure, regime shifts, and global teleconnections over the same period are examined in a companion study (Tebbaai et al., 2026). Because the SaOI represents the pressure contrast between the subtropical Atlantic and Saharan circulation systems, establishing its temporal characteristics is an important prerequisite for understanding its potential relationships with regional hydroclimatic and atmospheric variability. The index construction follows established methodologies employed for the NAOI(Hurrell, 1995; Jones et al., 1997), AO (Thompson & Wallace, 2001), and Southern Annular Mode (Gong & Wang, 1999), utilizing normalized pressure differences to ensure comparability across years, with seasonal anomalies obtained separately by removing the calendar-month climatological cycle when required (Hurrell, 1995; Thompson & Wallace, 2001).

The present study establishes the statistical and temporal characteristics of the Saharan Oscillation Index (SaOI) over the 1950–2024 period. Rather than assuming that the SaOI represents a persistent low-frequency climate oscillation, we systematically assess its distributional properties, temporal persistence, spectral structure, and long-term variability using complementary time- and frequency-domain diagnostics. Specifically, we evaluate its statistical distribution and variability, quantify temporal memory through autocorrelation and partial autocorrelation analyses, identify dominant timescales using Fourier and continuous wavelet transforms, and assess long-term trends using the Mann-Kendall test and Sen’s slope estimator. Particular emphasis is placed on distinguishing the persistent climatological seasonal cycle from non-seasonal short-term variability and determining whether apparent interannual variability represents coherent oscillatory behavior or episodic fluctuations. This statistical baseline provides a reproducible foundation for subsequent investigations of SaOI teleconnections and regional climate impacts.

The paper is organized as follows. Section 2 describes data sources and analytical methodologies. Section 3 presents statistical characterization including distribution assessment and examination of variability over time. Section 4 examines temporal persistence through autocorrelation and partial autocorrelation analyses, distinguishing seasonal persistence from residual non-seasonal short-term dependence. Section 5 presents spectral analyses via Fourier transform and continuous wavelet transform, identifying dominant timescales of variability. Section 6 analyzes long-term trends using robust non-parametric methods. Section 7 synthesizes findings, discusses physical interpretations and comparisons with established indices, and outlines implications for understanding Northwest African atmospheric dynamics.

## DATA AND METHODS

This study examines the temporal behavior and intrinsic variability of the (SaOI) over the 1950–2024 period through comprehensive time-series diagnostics. The analysis includes statistical characterization, distribution assessment, persistence evaluation, spectral decomposition, and trend detection to identify dominant periodicities and long-term trends in the (SaOI). These methodologies follow standard practices established for atmospheric circulation indices such as the North Atlantic Oscillation (Hurrell, 1995; Jones et al., 1997), and Arctic Oscillation (Thompson & Wallace, 2001).

The analysis uses monthly Saharan Oscillation Index (SaOI) values for 1950–2024, derived from monthly mean sea-level pressure (SLP) fields from the ERA5 reanalysis (Hersbach et al., 2020). The 75-year record provides a consistent basis for evaluating the distributional properties, temporal dependence, spectral characteristics, and long-term variability of the index.

### The (SaOI)

The Saharan Oscillation Index (SaOI) was calculated from sea-level pressure (SLP) derived from daily ERA5 reanalysis data at 0.25° spatial resolution (Hersbach et al., 2020) over 1950–2024. SLP was extracted at Ponta Delgada, Azores (37.79°N, 25.5°W), representing the Azores High, and Niamey, Niger (13.51°N, 2.10°E), representing the Saharan low-pressure center (Fig. 1). Following Khomsi et al. (2020), the SaOI was defined as the difference between the normalized pressures at these two locations:

$${\mathrm{SaOI}}_{d}=P_{\mathrm{Azores},d}^{*}\;-\;P_{\mathrm{Niamey},d}^{*}$$

where the normalized pressure at each location was calculated as

$$P_{d}^{*}=\frac{P_{d}\;-\;\bar{P}}{\sigma_{P}}$$

where $P_{d}$ is daily SLP, while $\bar{P}$ and $\sigma_{P}$ are the mean and standard deviation of SLP calculated over the full 1950–2024 period at the corresponding location. This normalization places the two-pressure series on a common dimensionless scale while preserving their temporal and seasonal variability. The mean seasonal cycle was removed separately, when required, by subtracting the corresponding calendar-month climatological mean from the monthly SaOI series. This procedure parallels the derivation of the NAOI (Hurrell, 1995), AOI (Thompson & Wallace, 2000), and Southern Annular Mode (Gong & Wang, 1999), which also express normalized pressure or height differences between key centers of action.

For analyses that require separating seasonal and non-seasonal variability, the monthly SaOI climatology was calculated for each calendar month over 1950–2024. Monthly SaOI anomalies were obtained by subtracting the corresponding calendar-month climatological mean from each monthly SaOI value. The original monthly SaOI series was retained to characterize the seasonal cycle, whereas the deseasonalized series was used for analyses of persistence and non-seasonal variability.

In the subsequent analyses, Monthly SaOI values were calculated as arithmetic means of the available daily SaOI values within each calendar month. The original monthly SaOI series was retained for phase classification and characterization of the climatological seasonal cycle. For analyses requiring separation of seasonal and non-seasonal variability, a calendar-month climatology was calculated over 1950–2024, and monthly SaOI anomalies were obtained by subtracting the corresponding calendar-month climatological mean from each monthly value. The deseasonalized series was used for persistence and non-seasonal variability analyses, including ACF, PACF, and spectral analysis of interannual-to-decadal variability.

### Statistical, Persistence, and Spectral Analysis

The statistical properties of the (SaOI) were characterized using mean, variance, skewness, and kurtosis (Wilks 2011). Distributional diagnostics included histograms, Gaussian kernel density estimation (Silverman, 1986), box plots, and Q-Q plots to assess normality and identify outliers (Tukey, 1977).

Temporal persistence was evaluated using the autocorrelation function (ACF) and partial autocorrelation function (PACF) applied to the deseasonalized monthly SaOI series. To assess persistence independently of the climatological seasonal cycle, the monthly SaOI series was deseasonalized by subtracting the corresponding 1950–2024 calendar-month climatological mean. The ACF was calculated over successive monthly lags to quantify the persistence and decay of temporal dependence, while the PACF was used to characterize the dependence structure after accounting for correlations at shorter lags. Lag-1 autocorrelation (r_1_) was used as a measure of short-term persistence. The deseasonalized series yielded r_1_ = 0.128, indicating weak residual month-to-month dependence after removal of the seasonal cycle. The e-folding timescale was not interpreted as a measure of persistence because the deseasonalized series did not exhibit a dominant AR(1) structure in the PACF.

Spectral behavior was examined using Fast Fourier Transform (FFT) and Continuous Wavelet Transform (CWT) techniques. The FFT was first applied to the original monthly SaOI series, after removal of its overall temporal mean, to characterize dominant periodic components, including annual and subannual variability. A separate FFT analysis was performed on the deseasonalized monthly SaOI series, obtained by removing the corresponding calendar-month climatological means, to isolate non-seasonal interannual-to-decadal variability. For time-frequency analysis, the CWT was applied to the original monthly SaOI series, thereby retaining its climatological seasonal cycle. The CWT used a complex Morlet wavelet (ω_0_ = 6), and wavelet power was calculated as the squared modulus of the wavelet coefficients and expressed on a log₂ scale. Periods from 2 to 96 months were examined. Statistical significance of local wavelet power was assessed against an AR(1) red-noise background. An AR(1) coefficient of 0.1571 estimated from the deseasonalized series was used to define the red-noise background for wavelet significance testing. Local wavelet power was evaluated at the 95% and 99% significance levels, while the global wavelet spectrum confirms a dominant peak near 12 months that lies well above the 95% AR(1) background level.. The cone of influence (COI) was used to identify regions affected by edge effects. The original monthly series was therefore retained for the CWT to characterize the temporal stability of the seasonal cycle, whereas the deseasonalized series was used to characterize non-seasonal persistence and to estimate the AR(1) background (Bloomfield 2000; von Storch & Zwiers 1999). Spectral power was expressed as a function of period, with frequencies converted to months for interpretation (Grinsted et al., 2004; Torrence & Compo 1998).

Long-term monotonic trends were assessed using the non-parametric Mann–Kendall (MK) test (Mann 1945; Kendall 1975). To account for serial dependence, the effective sample size (ESS) was estimated following Yue & Wang (2004), using the lag-1 autocorrelation of the linearly detrended series to adjust the variance of the MK statistic. Sen’s slope estimator was used to quantify the magnitude of the monotonic trend. Statistical significance was assessed at p < 0.05. Detailed mathematical formulations of the trend analysis are provided in Supplementary Table S1.

## RESULTS AND DISCUSSION

### Temporal Evolution and Statistical Characterization of the (SaOI)

The 75-year monthly (SaOI) time series (1950–2024), (Fig. 2) reveals a dominant positive mean (μ ≈ 0.1954) and substantial month-to-month variability (~0.10–0.15 standardized units). Negative anomalies are relatively rare (~12 % of months), whereas strong positive values (SaOI > 0.45) occur more frequently (~27 %), particularly during boreal spring. This seasonal clustering is consistent with enhanced meridional pressure gradients between the Azores High and Saharan Low and the associated subsidence mechanism described by Rodwell & Hoskins (2001), together with the climatological position of the descending Hadley branch.

A weak positive long-term trend is evident in the SaOI, with a Sen’s slope of approximately +0.00041 units yr^−1^. However, after accounting for serial dependence using the Yue–Wang effective-sample-size correction, the trend is not statistically significant (p ≈ 0.17). The corresponding ordinary least-squares trend is +0.000412 units yr^−1^ for the monthly series and +0.000428 units yr^−1^ for the annual mean series (Fig. 2). Over 1950–2024, the fitted trend corresponds to an increase of approximately 0.03 index units (~0.26σ), while the trend explains less than 1% of the total variance. Thus, the long-term increase is small relative to the pronounced month-to-month and interannual variability. This weak positive trend is physically consistent with reported changes in the Hadley circulation and subtropical pressure systems, although attribution of the SaOI trend to anthropogenic forcing is beyond the scope of this study (Iqbal et al., 2019; Kang & Polvani, 2011; Tandon et al., 2013). Although multidecadal modulations exist, including elevated SaOI values during the 1970s–1980s and weaker amplitudes in the early 2020s, no visually apparent abrupt shifts in variance are evident in the record.

After removal of the climatological seasonal cycle, the SaOI exhibits weak short-term persistence (Fig. 3). The lag-1 autocorrelation is r_1_ = 0.128, indicating only modest month-to-month dependence in the deseasonalized series. The ACF decreases rapidly toward zero with increasing lag, while the PACF does not exhibit a dominant lag-1 structure characteristic of a strong AR(1) process. These results indicate that much of the apparent persistence in the original monthly series is associated with the recurring seasonal cycle, whereas the remaining non-seasonal SaOI variability exhibits relatively weak short-term memory.

### Statistical Distribution and Variability Characteristics

The main distributional properties of the SaOI series are summarized in Table 1. The monthly SaOI distribution is approximately Gaussian in its central range, with modest positive-tail asymmetry, centered at μ = 0.1954 with σ = 0.1137 (Fig. 4a). The mean and median (0.1921) differ by < 0.003, confirming a symmetric central tendency with minimal outlier influence. Mild positive skewness (+ 0.15) indicates a slight preference for strong-gradient months (SaOI > 0.45) relative to weak-gradient months (SaOI < 0). This right-tail elongation reflects the climatological bias toward enhanced Azores-High and Saharan-Low pressure contrasts, particularly during spring, when land-sea thermal gradients peak.

The kernel density estimate (Fig. 4b) exhibits a unimodal, near-Gaussian core with modest upper-tail heaviness and a truncated lower tail. Approximately 34 % of months exceed μ + 0.05, whereas only 22 % fall below μ − 0.05, consistent with a 2:1 dominance of positive anomalies. The physical interpretation of these deviations is based on established atmospheric dynamics which link intensified subtropical ridging to suppressed Sahel rainfall during positive (SaOI) values (Diakhaté et al., 2019; Evans et al., 2019; Nicholson & Webster, 2007).

Box-plot diagnostics (Fig. 4c) indicate an interquartile range (IQR = 0.152; Q_1_ = 0.113; Q_3_ = 0.265) nearly identical to the theoretical normal expectation (IQR/σ = 1.34 vs 1.35). Extreme positive anomalies (SaOI > 0.47) are slightly more frequent than negative ones, corresponding to physically realistic episodes of pronounced Azores-High amplification (May 1956) and rare weak-gradient winters (e.g., December 1961).

The Q-Q plot (Fig. 4d) confirms near-linearity for the central 90 % of quantiles, with only slight positive-tail deviation (observed 99th percentile = 0.48 vs theoretical 0.46). Formal tests (Shapiro-Wilk W = 0.996, p = 0.018; Anderson-Darling A^2^ = 1.32, p = 0.006) detect statistically significant but small departures from normality. Given the near-linearity of the central quantiles and the small magnitude of skewness, the Gaussian distribution provides a reasonable descriptive approximation, although strict normality is rejected by the formal tests.

The near-Gaussian central distribution provides a useful descriptive approximation, although the formal tests indicate statistically significant departures from normality. The slightly heavier positive tail may reflect the asymmetric climatological behavior of the Azores-Sahara pressure gradient, although the specific physical mechanisms underlying the tail asymmetry cannot be established from the present statistical analysis.

Comparatively, the SaOI’s statistical shape resembles that of other large-scale atmospheric indices such as the North Atlantic Oscillation (NAO; Jones et al. 1997) and the Arctic Oscillation (AO; (Thompson & Wallace, 2001)), which exhibit weak skewness and stochastic variability. This contrasts with coupled modes like ENSO, whose strong positive skewness (+ 0.5 – 1.0) results from nonlinear ocean-atmosphere feedbacks (Burgers & Stephenson, 1999). The SaOI’s weak asymmetry and broadly consistent amplitude variability are compatible with a predominantly atmospheric character, while external climate influences may contribute to its interannual modulation.

### Frequency-domain structure (FFT analysis)

The Fast Fourier Transform (FFT) spectrum (Fig. 5a–b) highlights a dominant annual harmonic at f = 0.083 month^−1^ (T = 12 months), explaining ≈ 68 % of total variance, and a secondary semi-annual peak (f = 0.167 month^−1^; T = 6 months) contributing ≈ 12 %. These peaks represent recurrent seasonal variability associated with the annual cycle of solar heating, land–sea thermal contrast, and seasonal migration of subtropical circulation, and should therefore be interpreted as deterministic seasonal structure rather than as independent low-frequency oscillatory modes.

At higher frequencies (T < 5 months), spectral power is relatively weak and broadband, consistent with non-periodic atmospheric variability. (Blackmon 1976). At low frequencies (f < 0.05 cycles month^−1^; T > 20 months), spectral power forms a broad continuum rather than distinct narrow-band peaks. This low-frequency background indicates weaker, broadband interannual-to-decadal variability rather than evidence for a discrete oscillatory mode (Delworth & Mann, 2000; Hasselmann, 1976; von der Heydt et al., 2021). No distinct narrow-band interannual or decadal peaks are evident, indicating that SaOI variability is dominated by fast atmospheric processes rather than discrete oscillatory modes.

### Time-frequency structure (wavelet analysis)

To examine the temporal stability of the dominant periodicities, the Morlet CWT was applied to the original monthly SaOI series (Fig. 6a) (Torrence & Compo 1998). A pronounced band centered near 12 months extends across most of the 1950–2024 record and predominantly exceeds the 99% significance level outside the cone of influence. The global wavelet spectrum independently confirms a dominant peak near 12 months that clearly exceeds the 95% AR(1) significance threshold (Fig. 6b). These results demonstrate that the annual cycle is the most persistent and statistically robust timescale in the SaOI.

A weaker semi-annual component near 6 months is also evident, but significant power occurs intermittently rather than continuously throughout the record. In contrast, variability at periods longer than one year is less temporally coherent, appearing as episodic enhancements rather than as a persistent band. These longer-period features should therefore not be interpreted as evidence of a self-sustained interannual oscillation. Because the present analysis is restricted to periods up to 96 months and long-period power is increasingly affected by the cone of influence, interpretation of variability approaching the upper period limit remains cautious.

### Global wavelet spectrum and variance partitioning

The Global Wavelet Spectrum (GWS; Fig. 6b) corroborates the FFT results, showing its strongest and statistically significant concentration near the annual timescale, with a weaker secondary maximum near six months. Beyond the dominant seasonal band, wavelet power decreases substantially and does not form a persistent narrow-band interannual peak.

Beyond the dominant seasonal band, the wavelet analysis shows episodic rather than persistent interannual variability. No coherent interannual oscillatory band is evident within the reliably interpretable period range of the analysis. Variability approaching the upper period limit should be interpreted cautiously because of the increasing influence of the cone of influence.

This behavior parallels that of the NAO and AO (Hurrell & Deser, 2009; Thompson & Wallace, 2001) and supports viewing the SaOI as a regional manifestation of stochastic atmospheric variability over North Africa.

### Discussion and Climatic Implications

#### Dynamical Interpretation of SaOI Variability

The SaOI represents the pressure contrast between the Azores High and the Saharan Low, encapsulating the strength and position of the subtropical Hadley circulation over North Africa. The combined time- and frequency-domain evidence supports interpreting the SaOI as a diagnostic of regional atmospheric pressure variability dominated by the climatological seasonal cycle, with relatively weak non-seasonal short-term persistence. The dominant 12-month and 6-month spectral components, accounting for approximately 68% and 12% of the total spectral variance, respectively, indicate strong recurring subannual periodicity in the SaOI.

After removal of the climatological seasonal cycle, the SaOI exhibits weak short-term persistence (r_1_ = 0.128), indicating that much of the apparent persistence in the original monthly series is associated with the recurring seasonal cycle. The absence of a dominant lag-1 PACF structure further argues against interpreting the deseasonalized series as a strong AR(1) process. This behavior is broadly consistent with stochastic atmospheric variability characterized by red-noise-like persistence (Delworth & Mann, 2000; Hasselmann, 1976; Mitchell, 1976). Similar persistence characteristics have been reported for other atmospheric modes, including the North Atlantic Oscillation (NAO) and Arctic Oscillation (AO), which exhibit substantial short-term variability and red-noise-like behavior (Hurrell & Deser, 2009; Thompson & Wallace, 2001).

#### Links to Large-Scale Circulation and Teleconnections

The SaOI exhibits a weak positive long-term trend, but this trend is not statistically significant after accounting for serial dependence (p ≈ 0.17). This tendency is physically consistent with broader changes in subtropical atmospheric circulation reported in the literature, including poleward expansion of the Hadley circulation and changes in subtropical ridge strength. However, the present analysis does not establish a causal attribution of the SaOI trend to anthropogenic climate change (Iqbal et al., 2019; Kang & Polvani, 2011). A strengthening Azores High enhances subtropical subsidence and shifts the ITCZ northward, consistent with the expanding subtropical dry zone (Nicholson, 2018; Scheff & Frierson, 2012).

Interannual variability appears episodic rather than coherently oscillatory. Some episodes may overlap with periods of enhanced tropical climate variability, but establishing such relationships requires dedicated teleconnection analysis. Quantitative teleconnection results are detailed Tebbaai et al. (2026).

#### Climatic, Environmental, and Health Implications

The SaOI provides a physically interpretable indicator of the Azores-Sahara pressure gradient and associated subtropical atmospheric variability over North Africa. Periods of strong SaOI are physically consistent with stronger meridional pressure gradients and enhanced subsidence, conditions that can suppress convection and weaken monsoonal moisture transport, favoring warmer and drier conditions (Nicholson, 2013; Rodwell & Hoskins, 2001). Conversely, weaker SaOI states indicate reduced pressure gradients and are physically consistent with conditions favoring enhanced moisture transport and convective development over the Sahel (Evans et al., 2019; Nicholson, 2013). Stronger pressure gradients may also favor enhanced northeasterly flow and conditions conducive to Saharan dust uplift and transport, consistent with established aerosol-climate interactions (Prospero et al., 2020). Because dust and elevated temperature can affect air quality and human exposure, the SaOI may therefore have downstream environmental and public-health relevance. Quantifying these pathways requires dedicated analyses linking SaOI variability to aerosol, meteorological, and health datasets.

## CONCLUSION

This study provides a comprehensive statistical and spectral assessment of the SaOI over the 1950–2024 period, establishing its principal statistical, persistence, and spectral characteristics as a diagnostic of regional subtropical atmospheric pressure variability. Derived from the meridional sea-level pressure gradient between the Azores High and the Saharan-Sahel region, the SaOI exhibits a stable but predominantly positive mean state and moderate month-to-month variability, indicating that the index is dominated by atmospheric variability on the timescales resolved in this analysis, while weaker interannual modulation may reflect external climate influences. A weak positive long-term trend is present in the SaOI, but it is not statistically significant after accounting for serial dependence (p ≈ 0.17). The direction of the fitted trend is broadly consistent with reported changes in subtropical atmospheric circulation; however, the present analysis does not establish a causal attribution to anthropogenic climate change.

The statistical properties of the SaOI show a near-Gaussian central distribution with modest positive-tail asymmetry. Persistence diagnostics indicate that the moderate autocorrelation apparent in the original monthly series is substantially reduced after removal of the climatological seasonal cycle, yielding only weak non-seasonal short-term memory (r_1_ = 0.128). The wavelet red-noise background was characterized by an AR(1) coefficient of 0.1571. Fourier and wavelet analyses consistently identify the annual (~12-month) cycle as the dominant and most persistent timescale, while semi-annual (~6-month) variability is weaker and intermittent. Longer-period variability is episodic rather than coherently oscillatory within the reliably interpretable range of the analysis.

Climatologically, the SaOI encapsulates the dynamic coupling between the Azores High and the Saharan Low, providing a compact indicator of the strength of the Azores-Sahara pressure gradient and associated subtropical atmospheric variability over North Africa. Positive SaOI values indicate a stronger Azores-Sahara pressure gradient, physically consistent with enhanced subsidence and reduced moisture transport and therefore with conditions favorable for heat, drought, and dust activity. Conversely, negative values weaken the pressure gradient, allowing enhanced moisture transport and increased rainfall over the Sahel and adjacent regions. These linkages emphasize the SaOI’s value for understanding and monitoring regional hydroclimatic variability and atmospheric dust transport, which have far-reaching implications for agriculture, water resources, air quality, and public health.

Future research should extend this framework by investigating the contrasting dynamics of positive and negative SaOI phases and how SaOI variability modulates environmental extremes across the Sahara-Sahel region and the tropical Atlantic. Such analyses will enhance predictive understanding of heatwave frequency, drought intensity, and dust activity, providing actionable insights for regional adaptation strategies.

## Supplementary Material

Supplementary Methods

This table summarizes the analytical methods applied to the (SaOI) study, including key formulas and references.

## Figures and Tables

**Fig 1.**
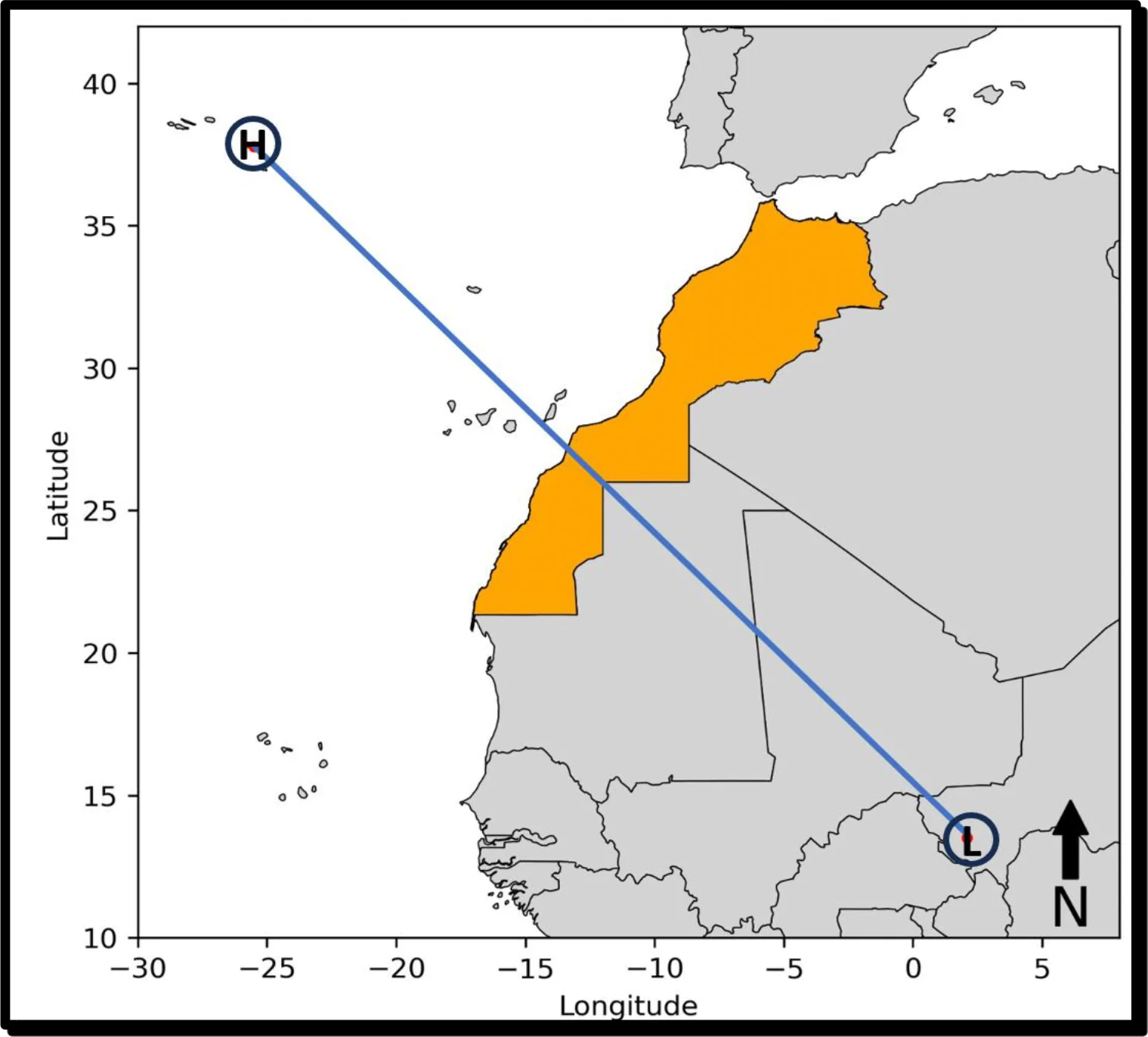
Definition of the (SaOI). Locations of the Azores High (H) near the eastern North Atlantic and the Saharan Low (L) over Niamey (Niger). SaOI is the standardized SLP difference H-L.

**Fig.2.**
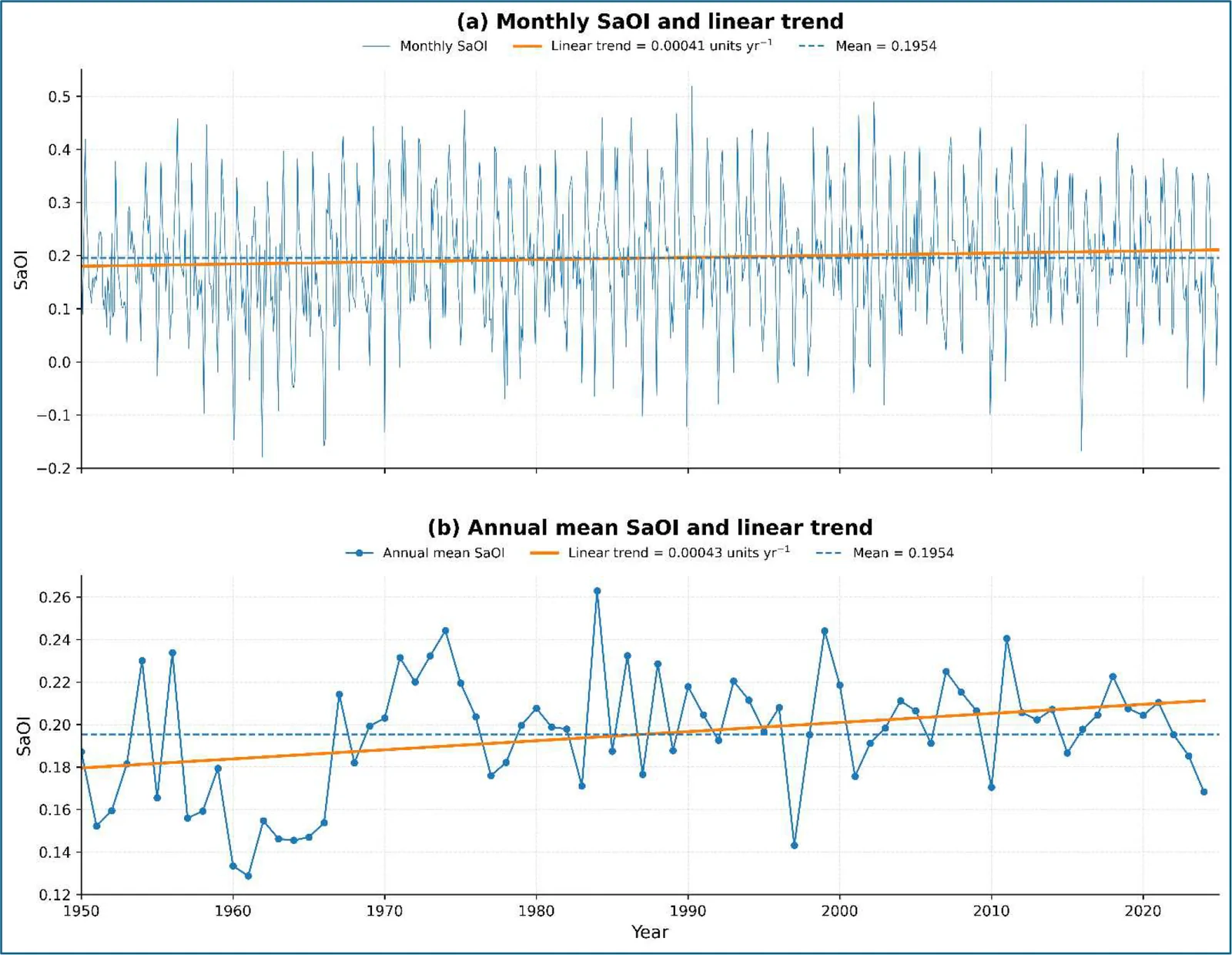
Long-term evolution of the Saharan Oscillation Index (SaOI), 1950–2024. (a) Monthly SaOI values with the climatological mean and an ordinary least squares (OLS) linear trend. (b) Annual mean SaOI with the corresponding mean and OLS linear trend. The OLS trends are shown for visualization, while statistical significance and robust trend magnitude were assessed using the modified Mann–Kendall test and Sen’s slope estimator.

**Fig 3.**
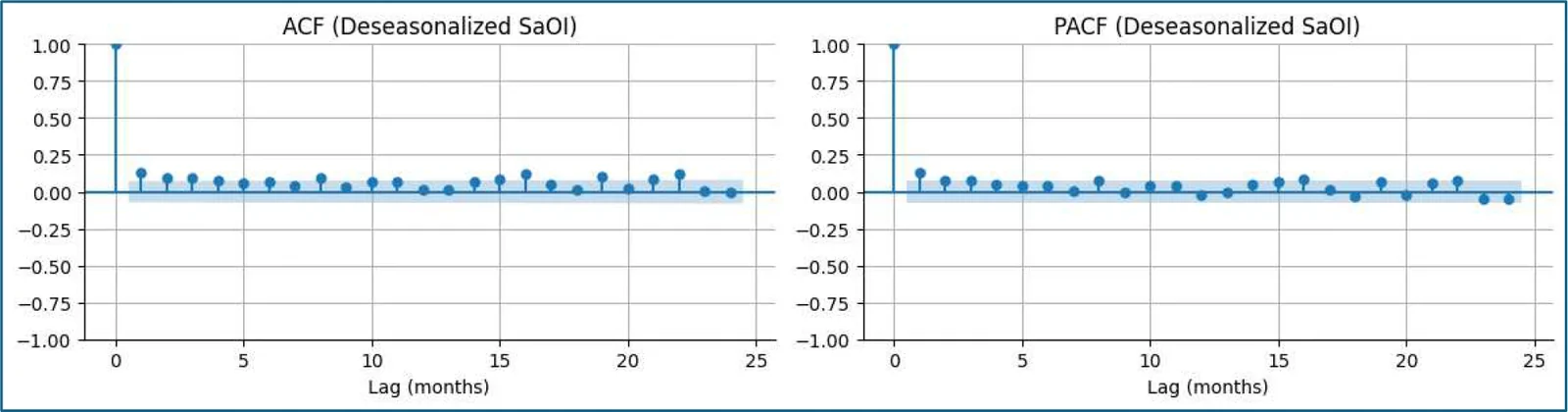
**Persistence diagnostics of the deseasonalized monthly SaOI (1950–2024)**, showing the autocorrelation function (ACF) and partial autocorrelation function (PACF). The lag-1 autocorrelation is r_1_ = 0.128, indicating weak residual short-term persistence after removal of the climatological seasonal cycle.

**Fig.4.**
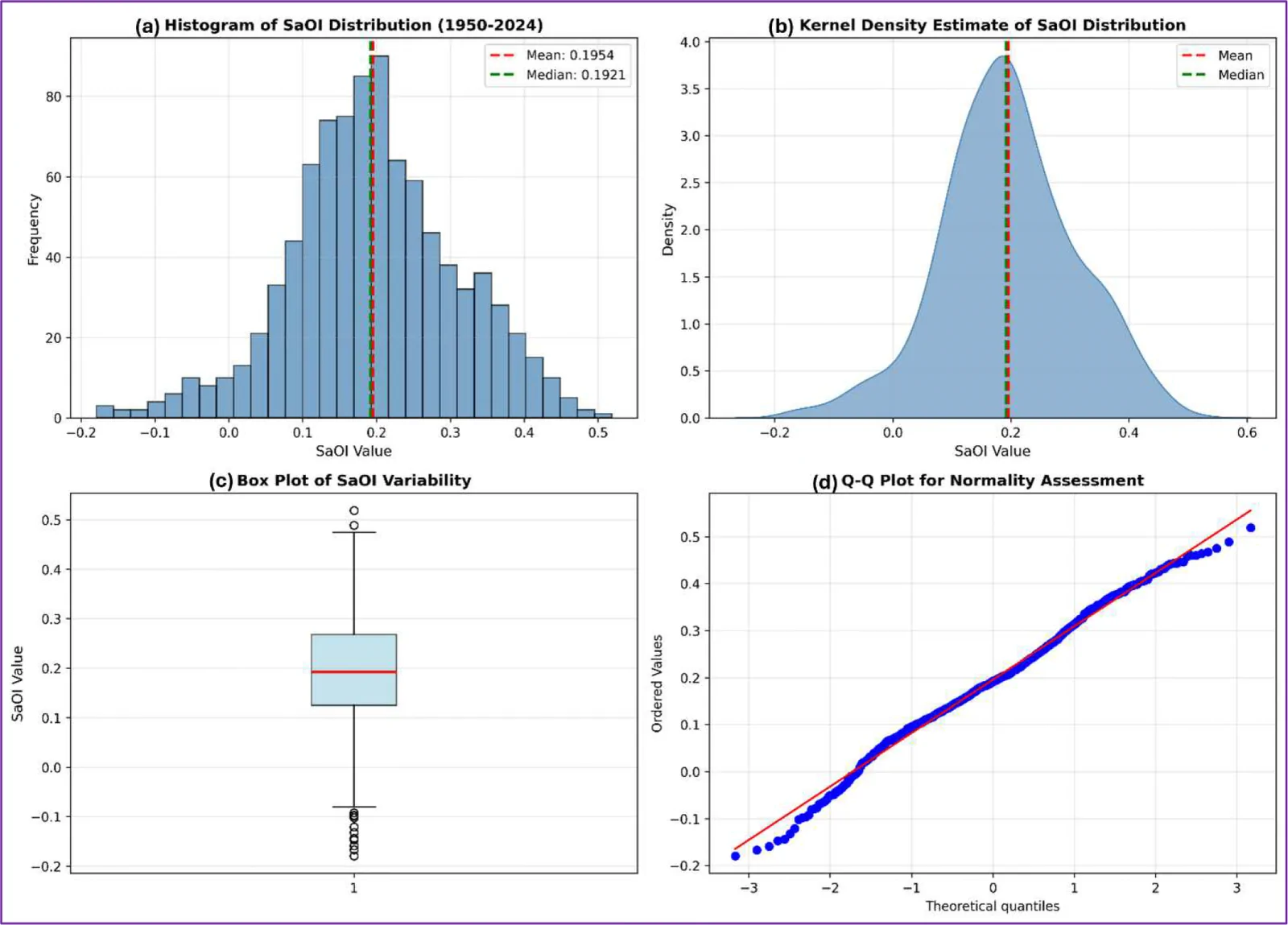
Distribution diagnostics. (a) Histogram; (b) KDE; (c) boxplot; (d) Q-Q plot versus normal.

**Fig 5.**
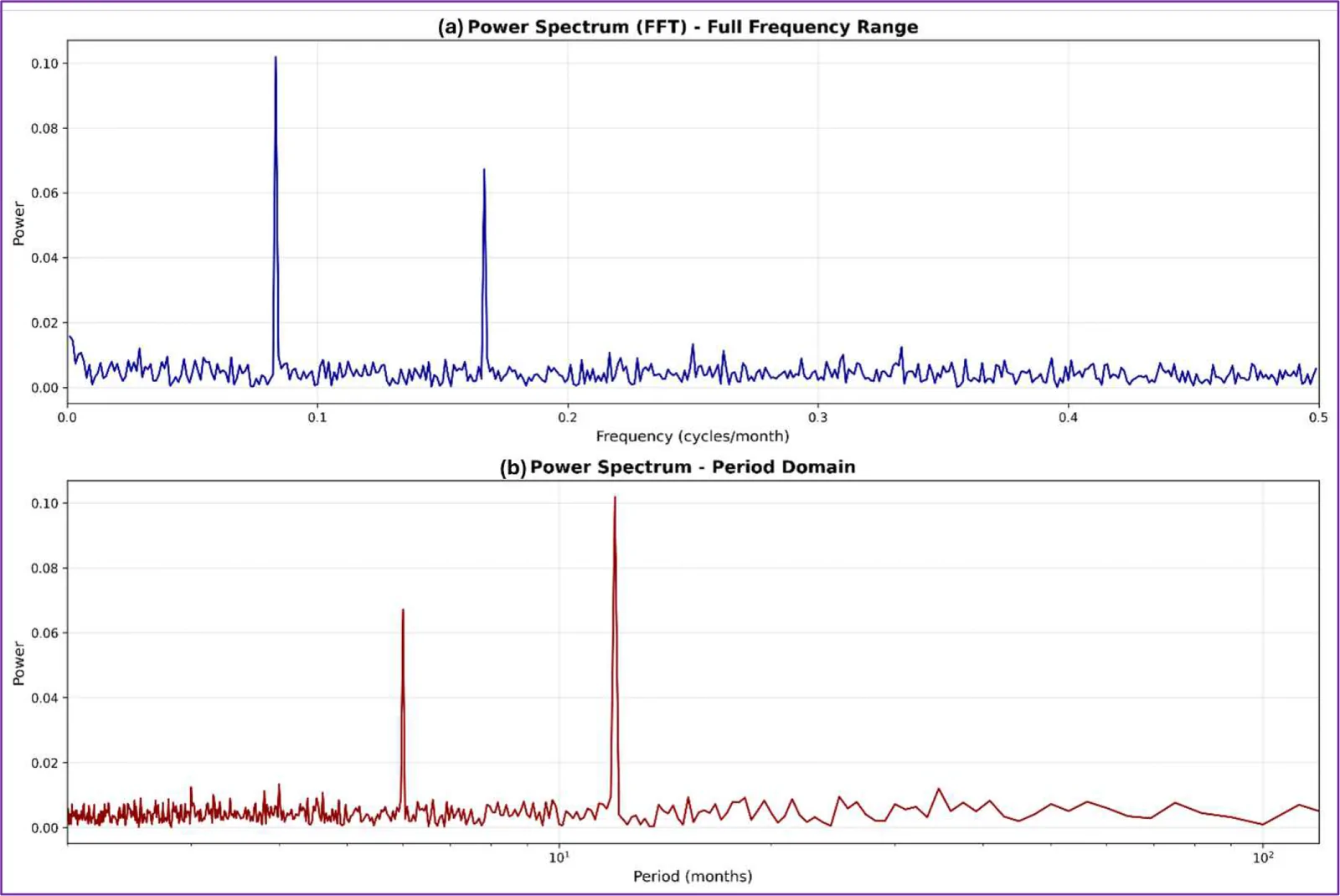
FFT power spectra of SaOI. (a) Frequency domain (0–0.5 cycles month^−1^); (b) period domain.

**Figure 6.**
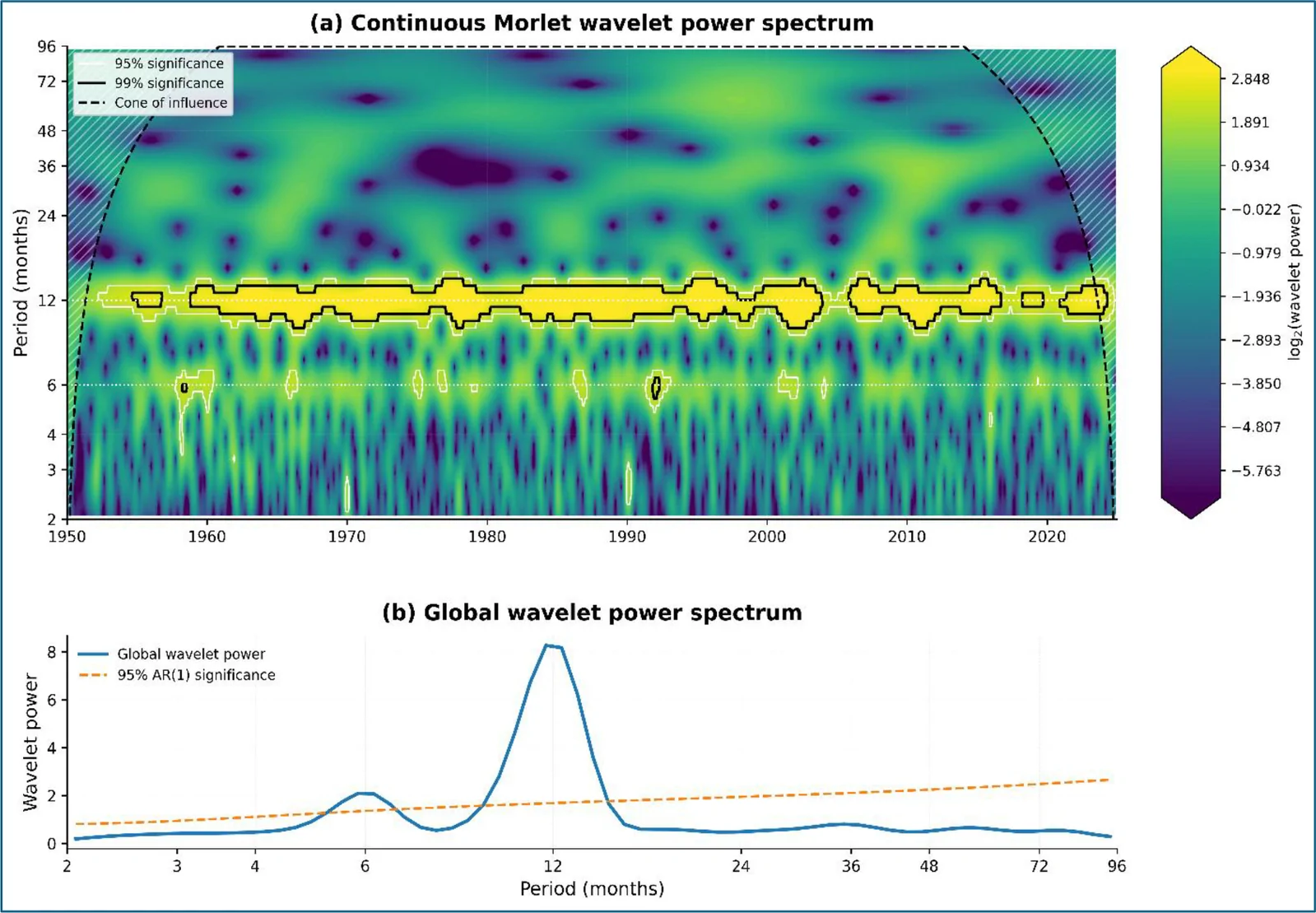
Continuous wavelet analysis of the Saharan Oscillation Index (SaOI), 1950–2024. **(a)** Morlet wavelet power spectrum of the standardized monthly SaOI series. White and black contours denote the 95% and 99% confidence levels, respectively, relative to an AR(1) red-noise background estimated from the deseasonalized SaOI series. The dashed boundary denotes the cone of influence, beyond which edge effects limit interpretation. Horizontal reference lines indicate the 6- and 12-month periods. **(b)** Global wavelet power spectrum and its 95% AR(1) significance level.

**Table 1.** Summary statistics of the SaOI for 1950–2024.

Section	Method Description & Formulae	Key References
1. Descriptive Statistics	Statistical moments: $\begin{array}{c} \mu =\frac{1}{n}\sum_{i=1}^{n}x_{i}\\ \sigma =\sqrt{\frac{1}{\left(n\;-\;1\right)}\sum_{i=1}^{n}{\left(x_{i}\;-\;\mu \right)}^{2}}\\ \gamma_{1}=\frac{1}{n}\sum_{i=1}^{n}{\left(\frac{x_{i}\;-\;\mu }{\sigma }\right)}^{3}\\ \gamma_{2}=\frac{1}{n}\sum_{i=1}^{n}{\left(\frac{x_{i}\;-\;\mu }{\sigma }\right)}^{4}\;-\;3 \end{array}$	Wilks (2011); Hurrell (1995); Thompson & Wallace (2001)
2. Histogram & KDE	Gaussian KDE: $f(x)=\frac{1}{nh}\sum_{i=1}^{n}K\left(\frac{x\;-\;x_{i}}{h}\right)$ Box-plot outliers: $x_{i}<Q_{1}-1.5\times IQR$ or $x_{i}>Q_{3}+1.5\times IQR$ Normality via Q-Q plots.	(Silverman (1986); Tukey, 1977; Wilk & Gnanadesikan (1968))
3. FFT Power Spectrum	Fourier decomposition: $\begin{array}{c} X(f)=\sum_{t=1}^{N}x(t)e^{-i2\pi ft}\\ P(f)=|X(f){|}^{2}\\ T=\frac{1}{f} \end{array}$ Identifies annual, interannual, and decadal variability.	Bloomfield (2000); von Storch & Zwiers (1999); Hurrell (1995)
4. Continuous Wavelet Transform	CWT: $\begin{array}{c} W(a,b)=\frac{1}{\sqrt{a}}\int_{-\infty }^{+\infty }x(t)\psi^{*}\left(\frac{t\;-\;b}{a}\right)\;dt\\ \psi (t)=\pi^{-1/4}e^{i\omega_{0}t}e^{-t^{2}/2} \end{array}$ Morlet wavelet ($\left(\omega_{0}=6\right)$) for time-frequency localization.	(Grinsted et al., 2004; Torrence & Compo 1998)
5. Mann-Kendall Trend Test	$\begin{array}{l} S=\sum_{i=1}^{n-1}\sum_{j=i+1}^{n}sgn\left(x_{j}-x_{i}\right)\\ sgn(\theta )=\left\{\begin{array}{ll} +1, & \text{if}\;\theta >0\\ 0, & \text{if}\;\theta =0\\ -1, & \text{if}\;\theta <0 \end{array}\right.\\ Var(S)=\frac{n(n\;-\;1)(2n\;+\;5)}{18}\\ Z=\left\{\begin{array}{ll} \frac{S\;-\;1}{\sqrt{Var(S)},} & \text{if}\;S>0\\ 0, & \text{if}\;S=0\\ \frac{S\;+\;1}{\sqrt{Var(S)},} & \text{if}\;S<0 \end{array}\right. \end{array}$ Modified MK adjusts Var(S) for autocorrelation.	Mann (1945); Kendall (1975); Yue & Wang (2004)

## Data Availability

The ERA5 reanalysis dataset used in this study is publicly available from the Copernicus Climate Data Store (https://cds.climate.copernicus.eu/). Processed SaOI data and supporting analysis scripts are available from the corresponding author upon reasonable request.
